# Survival Impacts of Perineural Invasion on Patients Under Different Radical Hysterectomies Due to Early Cervical Cancer

**DOI:** 10.3389/fonc.2022.889862

**Published:** 2022-04-29

**Authors:** Wei-wei Wei, Huihui Wang, Hong Zheng, Jiming Chen, Ru-xia Shi

**Affiliations:** Department of Gynecology, The Affiliated Changzhou No. 2 People’s Hospital of Nanjing Medical University, Changzhou, China

**Keywords:** perineural invasion, cervical cancer, nerve-sparing radical hysterectomy, laparoscopic surgery, survival impacts

## Abstract

**Objective:**

Cervical cancer is a common gynecological malignancy. In addition to the open radical hysterectomy (ORH) and laparoscopic radical hysterectomy (LRH), laparoscopic nerve-sparing radical hysterectomy (LNSRH) could be another treatment option since it could preserve urinary, colorectal, and sexual functions. However, LNSRH might result in early cancer metastasis and recurrence due to inadequate tumor resection. Additionally, whether LNSRH should be considered based on perineural invasion (PNI) status remains controversial. To assess different types of hysterectomy on the outcome of early cervical cancer with PIN.

**Methods:**

A retrospective study was performed in early cervical cancer patients who received ORH, LRH, or LNSRH between January 2012 and December 2019. Age, FIGO cancer stages, histopathological types, tumor size, histological grade, invasion depth, lymph node metastasis, lymphovascular space invasion, and PNI were documented. Disease-free survival (DFS) and overall survival (OS) were recorded.

**Results:**

A total of 174 patients were included, with 33, 69, and 72 patients received LRH, ORH, and LNSRH, respectively. Twenty-one patients (12.1%) had PNI. DFS (*P* = 0.000) and OS (*P* = 0.022) periods were shortened in positive PNI patients than in negative PNI patients (*P* = 0.000 and 0.022, respectively). In patients with positive PNI, lymph node metastasis, but not the surgery type, was an independent risk factor for DFS and OS (*P* = 0.000).

**Conclusion:**

Early cervical cancer patients with PNI had shorter postoperative DFS and OS periods. In these patients, lymph node metastasis, but not the type of hysterectomy, was independently associated with DFS and OS.

## Highlights

What is already known on this topic

PNI exists in early cervical cancer and is a poor prognostic factor for patients with early cervical cancer.

Effect of nerve-sparing radical hysterectomy in early cervical cancer with perineural invasion is unknown.

What this study adds

In early cervical cancer patients with perineural invasion, lymph node metastasis was a risk factor for survival.

How this study might affect research, practice or policy

Physicians should evaluate lymph node metastasis when treating early cervical cancer with perineural invasion.

## Introduction

Cervical cancer is a common gynecological malignancy globally (1). Its main treatment modalities include surgery and adjuvant chemoradiation therapy. Traditionally, early cervical cancer was treated with open radical hysterectomy (ORH) with or without bilateral pelvic lymphadenectomy. Studies have confirmed that ORH could prolong the disease-free survival (DFS) and the overall survival (OS) in patients with early cervical cancer (2). Lately, a minimally invasive surgical procedure, laparoscopic radical hysterectomy (LRH), has been introduced. Compared with traditional ORH, LRH could be a less traumatic procedure in early cervical cancer patients but might result in a lower DFS and OS (3–6). Both ORH and LRH could negatively impact the quality of life. Moreover, these patients frequently develop severe postoperative urinary, colorectal, and sexual dysfunctions (7). Consequently, a new type of surgery, nerve-sparing radical hysterectomy (NSRH), was proposed, preserving essential visceral nerves to maintain normal physiological functions Clinical trials have shown the benefits of NSRH in cervical cancer patients (8).

However, since NSRH leaves the visceral nerves and adjacent tissue intact, it raises concerns about incomplete cancer resection, early recurrence, and an increased risk of metastasis (9, 10). For example, a malignant mass can spread cancer cells contiguously along the nerve fibers, called perineural invasion (PNI) (11). PNI is recognized as one of the routes of metastatic spread of primary cancer (12). Studies have suggested that the presence of PNI is correlated with a poor prognosis in early-stage cervical cancer patients (13, 14). A shorter DFS and OS were reported after radical hysterectomy in cervical cancer patients with PNI than those without PNI (15). However, there is a paucity of studies exploring the outcome of NSRH in early cervical cancer patients with PNI.

This study investigated the impact on the treatment outcome of early cervical cancer with PNI with LRH, ORH, or LNSRH. We recorded and analyzed the DFS and OS to ascertain the postoperative prognosis and quality of life.

## Materials and Methods

### Study Design and Patient Selection

We conducted a retrospective study of histopathologically confirmed early cervical cancer patients who underwent surgical operations in Changzhou No. 2 People’s Hospital, affiliated with the Nanjing Medical University, between January 2012 and December 2019. The hospital Institutional Review Board approved this study.

The inclusion criteria were: 1) patients with a confirmed histopathological diagnosis of cervical adenosquamous carcinoma, squamous cell carcinoma, or adenocarcinoma, 2) patients with preoperative early cervical cancer stages I A2– II A2 based on the International Federation of Gynecology and Obstetrics (FIGO) staging system (2018) and 3) patients who had ORH, LRH, or LNSRH with additional pelvic lymphadenectomy +/- removal of paraaortic lymph nodes. Patients with incomplete medical records or abnormal vital organ function were excluded.

This study was approved by the hospital ethics committee (approval number [2021] YLJSD015). All procedures performed in the present study were in accordance with the ethical standards of the institutional and/or national research committee and with the 1964 Helsinki declaration and its later amendments or comparable ethical standards.

### Data Collection

We recorded the participant’s age and cervical cancer characteristics, including FIGO stage, pathological type, tumor size, histological grade, invasion depth, lymph node metastasis, lymphovascular space invasion (LVSI), and PNI.

All patients routinely had computed tomography (CT) of the abdomen and pelvis and/or magnetic resonance imaging (MRI) of the pelvis before surgery. These patients underwent ORH, LRH, or LNSRH with additional pelvic lymphadenectomy +/- removal of paraaortic lymph nodes. The indications for paraaortic lymphadenectomy included enlarged nodes on the CT or MRI examinations and a tumor diameter > 2 cm. Our LNSRH surgical procedure has been reported previously (16). In cervical cancer patients with signs of local extension, we tailored the surgical procedures to dissect the parametrial and paravaginal tissues. Since patients with parametrial tumor infiltration were not good candidates for the nerve-sparing procedure, we performed the partial nerve-sparing Kobayashi radical hysterectomy to preserve some portions of the inferior hypogastric plexus and the pelvic splanchnic nerves (9).

Postoperatively, patients received adjuvant radiotherapy if they had a risk of tumor recurrence. Risk factors for tumor recurrence included: 1) positive resection margins, parametrial invasion, and lymph node metastases; or 2) more than two of the following factors, including stromal invasion >1/2 of cervical wall thickness, tumor diameter > 4 cm, and LVSI (17–20).

**Figure 1:**
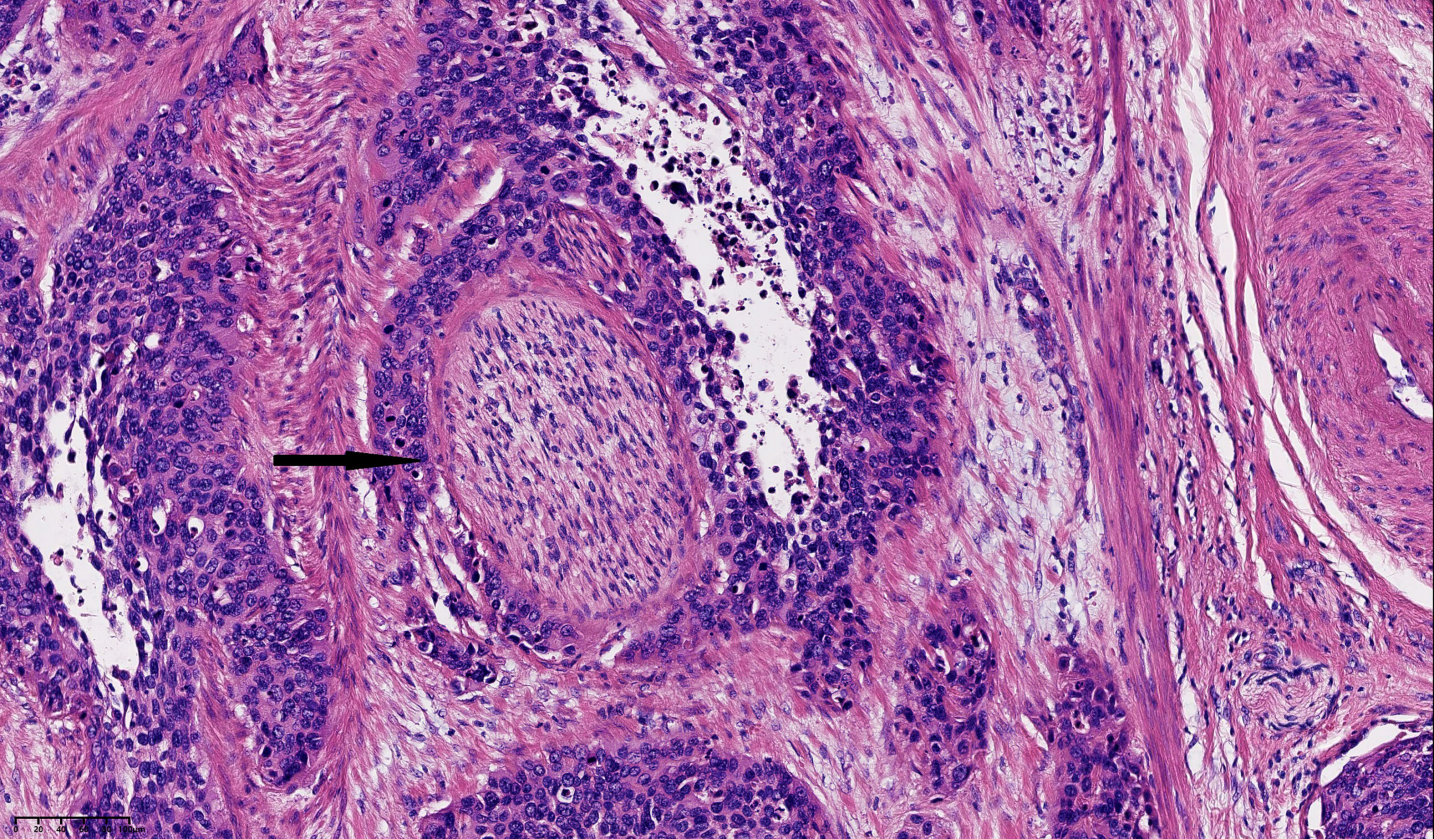
Histological image of perineural invasion (PNI) in cervical squamous cell carcinoma stained with Hematoxylin and eosin (H&E). PNI from small cancer cell clusters is indicated by arrows (magnification, x200). Scale bar: 20μm each grid.

Tumor tissue samples were obtained from the cervix and parametrial areas of invasion during surgery. They were fixed, embedded, and stained with hematoxylin-eosin (H&E). PNI was confirmed if the H&E staining showed that: 1) any layers of the nerve sheath, including the epineurium, perineurium, or endoneurium, were infiltrated by the cancer cells or 2) ≥ 33% of the nerve circumference was surrounded by the cancer cells (12, 21). An example of PNI is shown in **Figure 1**.

After the hospital discharge, patients were followed up by a telephone interview every three months. Follow-up information collected included general health status, cancer recurrence, and mortality. The duration of the follow-up was also documented.

DFS was the period from the time point of surgery to the timepoint of cancer recurrence identified on repeat CT or MRI. If there was no cancer recurrence, the time point of the last follow-up examination or death was used. Overall survival (OS) was the period from the time point of surgery to death.

### Statistical Analysis

Multivariate logistic regression analyses were conducted to examine the factors related to DFS and OS. Continuous data are shown as mean ± standard deviation (SD) and compared by one-way ANOVA. Categorical data are presented as percentages and compared by the Chi-square test. The Kaplan–Meier analysis was used to examine the DFS and OS. The log-rank test was used to calculate each corresponding *P*-value. Statistical significance was considered with a *P* < 0.05. All analyses were performed in SPSS 20.0 (SPSS, IBM, New York, USA).

## Results

### Participant Characteristic Comparisons

This study included 174 patients, with 33, 69, and 72 in the LRH, ORH, and LNSRH groups, respectively. We did not observe statistically significant differences in the FIGO stage, histopathological types, tumor size, histological grade, invasion depth, lymph node metastasis, and LVSI among the three groups (**Table 1**).

**Table 1:** Clinical and histopathological characteristics of study participants.

Characteristics	LRH group (N = 33)	ORH group (N = 69)	LNSRH group (N = 72)	*P*
Age, years (mean ± SD)	52.8 ± 8.0	52.8 ± 9.1	53.2 ± 10.2	0.085
FIGO stage, N (%)				0.920
IA	1 (3.0%)	0 (0.0%)	5 (6.9%)	
IB1	6 (18.2%)	14 (20.3%)	9 (12.5%)	
IB2	11 (33.3%)	15 (21.8%)	28 (38.9%)	
IB3	1 (3.0%)	9 (13.0%)	4 (5.5%)	
IIA1	2 (6.1%)	7 (10.1%)	9 (12.5%)	
IIA2	2 (6.1%)	5 (7.2%)	2 (2.8%)	
IIIC1	9 (27.3%)	18 (26.1%)	13 (18.1%)	
IIIC2	1 (3.0%)	1 (1.5%)	2 (2.8%)	
Pathology, N (%)				0.928
Squamous cell carcinoma	30 (90.9%)	61 (88.4%)	64 (88. 9%)	
Adeno/adenosquamous carcinoma	3 (9.1%)	8 (11.6%)	8 (11.1%)	
Histological grade, N (%)				0.089
Well-differentiated	8 (24.2%)	14 (20.3%)	14 (19.4%)	
Moderately differentiated	17 (51.5%)	33 (47.8%)	29 (40.3%)	
Poorly differentiated	8 (24.2%)	22 (31.9%)	29 (40.3%)	
Tumor size, cm (mean ± SD)	3.5 ± 1.1	3.8 ± 1.2	3.5 ± 1.3	0.642
Depth of invasion				0.274
<1/2	13 (39.4%)	24 (34. 8%)	30 (41. 7%)	
≥1/2	20 (60.6%)	45 (65.2%)	42 (58.3%)	
LVSI				0.330
Present	9 (27.3%)	26 (37.7%)	29 (40.3%)	
Lymph node metastasis				
Positive	10 (30.3%)	19 (27.5%)	15 (20.8%)	0.246
Perineural invasion				
Positive	2 (6.1%)	10 (14.5%)	9 (12.5%)	0.407
Rejected adjuvant radiation therapy, N (%) *	3 (14.3%)	6 (12.0%)	3 (5.7%)	0.152
Cancer recurrence	0 (0.0%)	4 (66.7%)	1 (33.3%)	

LVSI, lymphovascular space invasion.

*These patients were considered to have the risk of tumor recurrence and were offered the adjuvant radiation therapy. However, they refused the treatments.

A total of 21 patients (12.1%) had PNI, with 2, 10, and 9 patients in the LRH, ORH, and LNSRH groups, but with no significant difference among them.

Postoperatively, 112 patients received adjuvant radiation therapy. Twelve patients rejected radiotherapy, with three in the LRH, six in the ORH, and three in the LRH group. Five of these 12 patients had cancer recurrences, with four in the ORH group, and one in the LNSRH group.

### Comparisons of Postoperative Survival Among the Three Groups

Twelve patients were lost to follow-up, while the remaining162 patients had a median postoperative follow-up time of 44.9 months (0 – 115 months).

**Figure 2:**
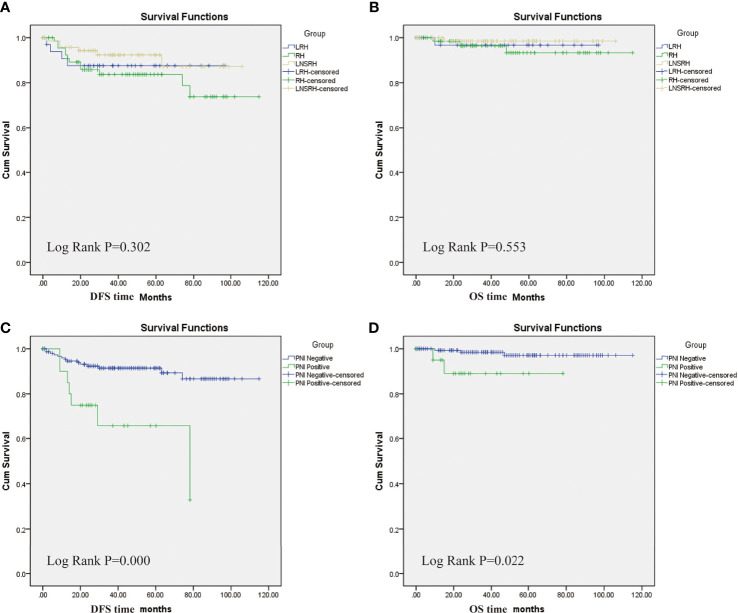
Kaplan-Meier survival analyses. **(A)** Comparisons of disease-free survival (DFS) periods among three groups. **(B)** Comparisons of overall survival (OS) periods among three groups. There was no statistically significant difference in DFS and OS among the three groups. **(C)** DFS periods between positive and negative PNI groups. **(D)** OS periods between positive and negative PNI groups. PNI positive group had lower DHS and OS than the PCI negative group.

The DFS rates were 87.9, 82.6, and 91.7%, and the OS rates were 97.0, 95.7, and 98.6% in the LRH, ORH, and LNSRH groups, respectively, with no statistically significant difference among the three groups (*P* = 0.302 for DFS and 0.553 for OS, **Figures 2A, B**).

Patients were assigned into either the PNI positive or PNI negative groups. The PNI positive and PNI negative groups had DFS rates of 66.7 and 90.8% and OS rates of 90.5 and 98.0%, respectively. Positive PNI patients had significantly lower DFS (*P* = 0.000) and OS (*P* = 0.022) than the negative PNI patients (**Figures 2C, D**).

### Multivariate Regression Analyses

In 21 patients with positive PNI, multivariate logistic regression analysis revealed that lymph node metastasis, but not the type of surgery, was independently correlated with DFS and OS (*P* = 0.000) (**Table 2**).

**Table 2:** Multivariate analysis for disease-free survival and overall survival.

Variables	Disease-free survival time	Overall survival time
	*P*	*P*
FIGO stage, n (%)	0.216	0.258
Depth of invasion	0.815	0.250
LVSI	0.652	0.146
Lymph nodes metastases	0.000	0.000
Type of surgery*	0.990	0.690

LVSI, lymphovascular space invasion.

*Types of surgery, open radical hysterectomy, laparoscopic radical hysterectomy, or laparoscopic nerve-sparing radical hysterectomy.

## Discussion

Despite increased prevention and screening efforts, cervical cancer is still a common gynecological malignancy in women, with more than 300,000 deaths worldwide annually (1). Its management should consider the postoperative survival chance and the quality of life.

Surgical resection is the mainstay of treatment in early cervical cancer patients. ORH is the traditional surgical approach and was confirmed to improve the survival chance in cervical cancer patients. Recently, LRH was introduced as a new surgical modality, and early studies showed the benefits of LRH, including less trauma, fast recovery, and short hospital stays (22). However, more and more studies have demonstrated inferior surgical outcomes, including short OFS and OS and early cancer recurrence (3–6). Our study did not observe any statistically significant differences in the baseline characteristics between patients with early cervical cancer who underwent either ORH or LRH. Patients treated with LRH or ORH had no statistically significant differences in the OS and DFS. More studies to investigate the surgical outcomes and survival in patients treated with these two types of surgery are required.

Both ORH and LRH could also cause postoperative bladder, colorectal, and sexual dysfunction, and decreased quality of life in affected patients due to physical discomfort and mental stress (7). NSRH was proposed to preserve the visceral nerves to retain postoperative pelvic functions (9). Clinical evidence has demonstrated that NSRH could be a practical and safe procedure, that may improve the postoperative quality of life of women with cervical cancer (23). However, whether the NSRH could affect the postoperative survival outcomes and cancer recurrence is still under investigation since cancer could spread along the splanchnic nerve, which might be inadequately removed by the NSRH (9, 10).

PNI refers to cancer cell invasion of the nerve fibers. Recent studies have shown that cervical cancer could have PNI. PNI was correlated with poor postoperative survival and cancer recurrence. In cervical cancer patients with PNI, NSRH might leave the cancer cells in the nerve fibers intact. In addition, studies have shown that PNI could be associated with other pathology risk characteristics and poor prognosis (15, 24). Cervical cancer patients with PNI could thus have a high risk for postoperative cancer recurrence or metastasis. Some authors suggested that PNI should be a major consideration when selecting NRSH for patients with cervical cancer (10). However, preoperative determination of PNI could be difficult. Studies have suggested that preoperative pelvic MRI imaging and intraoperative frozen section examinations might help to define the PNI, but the results were not conclusive. More studies are required to find the methods that can provide an accurate preoperative diagnosis of PNI.

The objective of our study was to ascertain whether different types of surgery could affect the prognosis of early cervical cancer with PNI. In our study, 12.1% of patients with early cervical cancer had PNI, which was more than 8.6% reported by Zhu et al. (13). This percentage difference might be because our patients had slightly higher FIGO stages than their patients. Our study results showed that early cervical cancer patients with PNI had lower postoperative DFS and OS than those without PNI. This result is consistent with previous reports (14, 15). Multivariate regression analysis demonstrated that, in all patients with PNI, the lymph node metastasis, but not the type of surgery, was an independent risk factor impacting the DFS and OS. This finding implied that LNSRH probably has similar efficacy as LRH and ORH in treating early cervical cancer patients with PNI. Therefore, physicians should carefully consider lymph node metastasis and not the type of surgery when considering hysterectomy in these patients with PNI. Lymph node metastasis and not the type of hysterectomy would determine the length of postoperative survival.

In this retrospective study, we performed NSRH laparoscopically. The minimally invasive techniques could enhance postoperative recovery, decrease postoperative complications, and improve short-term prognosis compared with major gynecologic surgery, such as RH (25, 26). We did not identify a statistically significant DFS and OS difference among the three groups, but the DFS and OS were higher in the LNSRH group than in the other two groups. These findings suggest that LNSRH is a feasible, safe, and effective surgical option for treating early cervical cancer with PNI (23).

Additional benefits of LNSRH could be the earlier return of bladder function, allowing patients to undergo earlier postoperative adjuvant radiotherapy, and potentially improving the chances of survival.

Our study’s strengths include its direct survival comparison among three different types of hysterectomy and relatively long-term follow-up periods. The study’s limitations include the retrospective design, single-center study, and a very small sample size. We only studied the survivals, but not the postoperative complications and quality of life of these patients. Future prospective studies should be conducted to validate our findings and address these questions.

In conclusion, patients with early cervical cancer and PNI had shorter postoperative DFS and OS periods. In these patients, lymph node metastasis, but not the surgery types, was independently associated with the DFS and OS.

## Data Availability Statement

The raw data supporting the conclusions of this article will be made available by the authors, without undue reservation.

## Ethics Statement

The studies involving human participants were reviewed and approved by Changzhou Second People’s Hospital Ethics Committee. The patients/participants provided their written informed consent to participate in this study.

## Author Contributions

W-WW: Data curation; Formal analysis; Funding acquisition; Investigation; Methodology; Writing - original draft. HW: Data collection; Methodology; Validation. HZ: Methodology; Validation; Software. JC: Resources; Supervision; Writing - review & editing. R-XS: Conceptualization; Methodology; Project administration. All authors contributed to the article and approved the submitted version.

## Funding

This work was supported by grants from the Changzhou Science & Technology Program (QN201931), the maternal and child health research project of Jiangsu Province ( F202138 ), the Scientific Research Support Program for Postdoctoral of Jiangsu Province(2019K064), and the Scientific Research Support Program for “333 Project” of Jiangsu Province (BRA2019161).

## Conflict of Interest

The authors declare that the research was conducted in the absence of any commercial or financial relationships that could be construed as a potential conflict of interest.

## Publisher’s Note

All claims expressed in this article are solely those of the authors and do not necessarily represent those of their affiliated organizations, or those of the publisher, the editors and the reviewers. Any product that may be evaluated in this article, or claim that may be made by its manufacturer, is not guaranteed or endorsed by the publisher.
